# Thrombopoietin and its receptor in normal and neoplastic hematopoiesis

**DOI:** 10.1186/s12959-016-0095-z

**Published:** 2016-10-04

**Authors:** Kenneth Kaushansky

**Affiliations:** School of Medicine, Stony Brook University, New York, NY 11784 USA

**Keywords:** Thrombopoietin, Hematopoiesis

## Abstract

Thrombopoietin was posited to exist in 1958 and cloned in 1994, and in the ensuing two decades we have learned a great deal about the physiology and pathology of the primary regulator of thrombopoiesis. This paper will review the role of the hormone and its receptor, the product of the c-Mpl proto-oncogene, in health and disease, including many unexpected effects in both normal and neoplastic hematopoiesis. Amongst these unexpected properties are a non-redundant effect on hematopoietic stem cells, a critical role in all three of the acquired, chronic myeloproliferative neoplasms, as well as both gain-of-function and loss-of-function mutations in congenital and acquired states of thrombocytopenia and thrombocythemia.

## Background

The term thrombopoietin first appeared in the literature in 1958 [1], to depict the primary regulator of platelet production. Over the ensuing 36 years numerous attempts to purify thrombopoietin were undertaken, but the identification of the orphan cytokine receptor, c-Mpl, in France in 1992 [2] catalyzed the successful cloning of the hormone by three groups in 1994 [3]. Since that time the role of thrombopoietin and c-Mpl has been evaluated extensively in normal and neoplastic hematopoiesis [4]. This paper will review that progress.

## Review

### The regulation of thrombopoietin

As the primary regulator of platelet production, it would be anticipated that the levels of thrombopoietin would be inversely related to blood platelet levels, and this is the case in most pathological conditions [5]. Patients with aplastic anemia have the highest levels of blood thrombopoietin (~1-2 ng/ml) with normal values of approximately 80 pg/ml. Patients with chemotherapy-induced thrombocytopenia have intermediate levels, although patients with immune thrombocytopenia have unexpectedly relatively normal levels. A number of physiological mechanisms account for these findings.

The liver is the largest single site of thrombopoietin production, producing the hormone in both constitutive and inducible fashion, a finding that helps to explain the thrombocytopenia seen in patients with hepatic failure. The baseline level of the hormone was thought to be mediated by receptor-medicated removal of the hormone from the circulation by platelets and/or marrow megakaryocytes. Platelets express low levels of c-Mpl, which take up thrombopoietin and degrade it [6]. This “sponging” of the hormone is thought to be an important regulatory “governor” of platelet production. More recently, this view has been challenged, with evidence that it is due to the sensing of senescent platelets, mediated by the hepatocyte Ashwell Morrell receptor [7], which is stimulated by desialated proteins. In addition to one or both of these mechanisms regulating platelet production, hepatocytes can be induced to produce additional thrombopoietin when stimulated by interleukin 6 [8], the mechanism of thrombocytosis seen in patients with chronic inflammatory conditions, such as ulcerative colitis or rheumatoid arthritis, the two most common causes of thrombocytosis.

Additional mechanisms of thrombopoietin production also exist; marrow stromal cells respond to thrombocytopenia with increased production of the hormone [9], and since the hormone acts in a paracrine fashion (directly adjacent), rather than hepatic endocrine hormone production (at a distance), one might assume that the marrow stromal cell production may play a particularly important role in platelet production.

### c-Mpl signal transduction

Once thrombopoietin binds to the c-Mpl receptor a number of signaling pathways are triggered. Thrombopoietin binds to the extracellular domain of c-Mpl, inducing dimerization of two copies of the receptor as the hormone displays two receptor binding sites on its four alpha helix tertiary fold, the A and D helix binding to the first receptor, and the A and C helices binding to the second receptor subunit [10]. Once the receptor dimer is formed, it allows the Jak2 signaling kinase bound to the membrane proximal region of the cytoplasmic domain to dimerize, allowing their cross phosphorylation (activation) [11] and subsequent phosphorylation of the membrane distal region of the cytoplasmic domain of the receptor and a number of proteins that tether to the newly generated phosphotyrosine sites of the receptor. Amongst the signaling pathways activated are the phosphoinositol-3-kinase [12], mitogen activated protein kinase [13] and several microRNA pathways [14]. The result is the alteration of a number of cell proliferation, cell survival and cell differentiation processes.

### Thrombopoietin and the hematopoietic stem cell

When initially defined as the primary regulator of thrombopoiesis, most assumed that the effects of thrombopoietin were restricted to megakaryocyte and platelet differentiation and maturation. The hormone was not supposed to stimulate the proliferation of megakaryocyte precursors, and definitely not support primitive hematopoietic cells at all. The availability of pure thrombopoietin has dispelled these incorrect first assumptions. Thrombopoietin is the most potent stimulator of megakaryocyte colony-forming cells [15], and is one of only two non-redundant hormones that affect hematopoietic stem cell survival and expansion (stem cell factor is the other). The stem cell effects of thrombopoietin can be demonstrated in vitro, with single cell culture experiments [16], in vivo in knock out mice (e.g. genetic elimination of *c-Mpl* or *THPO* reduce the number of transplantable hematopoietic stem cells by 10-fold [17]), and in people (missense or non-sense mutations of either gene cause congenital amegakaryocytic thrombocytopenia that can evolve into aplastic anemia if the mutation is severe [18]). Hence, c-Mpl is expressed in hematopoietic stem cells, which provides the basis for our, and others exploring the role of the cytokine and its receptor in diseases of the hematopoietic stem cell.

### Thrombopoietin, c-Mpl and the chronic myeloproliferative neoplasms

In 1950 William Dameshek postulated that the three chronic myeloproliferative neoplasms (then called syndromes), polycythemia vera, essential thrombocythemia and primary myelofibrosis were pathophysiologically related [19]. It took 55 years to provide a molecular explanation for this correct hypothesis. In 2005 four groups reported that virtually every patient with polycythemia vera, and about half of patients with essential thrombocythemia and primary myelofibrosis display one or two (exclusively P. vera patients) mutant alleles of the signaling kinase Jak2 [20–23]. The valine to phenylalanine at position 617 in the “pseudokinase” domain of Jak2 causes autonomous signaling. It has been postulated that the mutation changes the activation loop interaction with the kinase domain, although other mechanisms by which this point mutation might activate the kinase are being considered (e.g. spontaneous dimerization of the mutant Jak2), especially since other site mutations in the Jak2 kinase are also associated with/causative of myeloproliferative neoplasms.

Soon after description of the Jak2V617F mutation in the chronic myeloproliferative neoplasms, attention turned to non-Jak2V617F bearing diseases. A year later an activating mutation of the c-Mpl receptor was described, at position c-Mpl515 [24], which when transduced into marrow cells produced an aggressive myeloproliferative syndrome in animals. Overall, about 5–10 % of patients with essential thrombocythemia or primary myelofibrosis express this mutant form of the thrombopoietin receptor. A number of other patients were also shown to harbor mutations in a series of genes that affect epigenetic modifications of hematopoietic cells, such as Tet2 and several methylation modification enzymes. Altogether these mutations provide a molecular explanation for approximately 5–10 % additional patients with a non-Jak2V617F-bearing disease. And most recently, a full 30 % of patients lacking Jak2V617F display a truncation mutation of the endoplasmic reticulum chaperone and calcium buffering protein called calreticulin [25]. Elimination of the carboxyl terminus of this protein removes an endoplasmic reticulum retention signal, allowing the protein to travel to other sites in the cell.

Twenty years ago Li and colleagues described that one of the hallmarks of myeloproliferative neoplasms, spontaneous colony formation, was significantly reduced by reduction in expression of c-Mpl in marrow cells using an antisense oligonucleotide strategy [26]. This finding raises the question of whether c-Mpl might play an important role in *all* patients with acquired myeloproliferative neoplasms, and if so, what is it’s mechanism.

As described above, the signaling kinase Jak2 acts by binding to the cytoplasmic domain of c-Mpl and from there is dimerized when thrombopoietin binds to the receptor. So the question arose as to whether Jak2 binding to any cytokine receptor might serve as a scaffold to allow Jak2 dimerization. This question was addressed by our laboratory and others, and the conclusion is that any receptor that homodimerizes (as opposed to heterodimerization, as occurs with the IL-3 or GM-CSF receptor) can serve to support Jak2V617F-induced-growth factor independent cell growth [27]. Since the receptors for erythropoietin, granulocyte colony stimulating factor and thrombopoietin are all homodimers, theoretically Jak2V617F should be able to induce cytokine independent growth in cells expressing any of these three receptors. However, as mentioned above, that while c-Mpl is a bone fide stem cell receptor, neither the erythropoietin or the G-CSF receptors are expressed in hematopoietic stem cells.

In order to prove that c-Mpl is required for Jak2V617F induced myeloproliferative neoplasm, we and others have now created mouse models of myeloproliferation and crossed the mice with those null for c-Mpl. In our studies the expected rise in neutrophil and platelet counts, expansion of all types of hematopoietic colony forming cell types, splenomegaly and osteosclerosis were all eliminated in the Jak2V617F bearing mice that were also null for c-Mpl [28].

Finally, very recent data suggests that the myeloproliferative neoplasm induced by truncation mutations of calreticulin also require c-Mpl to induce disease; in this case, elimination of the endoplasmic reticulum tether allows calreticulin to bind to c-Mpl and travel to the cell membrane where the thrombopoietin receptor dimerizes and drives Jak2 activation [29].

## Conclusions

Since their cloning 22 years ago thrombopoietin and its receptor, the product of the c-Mpl proto-oncogene, have been extensively characterized, the physiology of hematopoiesis is now much better understood, small molecule receptor binding agonists produced and approved for clinical use, and the role of the hormone and receptor in human health and disease defined. Both congenital and acquired mutations of the hormone and its receptor cause either familial thrombocytosis and thrombocytopenia/aplastic anemia, depending on whether the mutations are loss of function or gain of function, and more recently the normal receptor was found to play a critical role in virtually all patients with chronic myeloproliferative neoplasms. These insights should lead to novel and improved therapeutic approaches to a range of disorders of hematopoiesis.

## Abbreviations

T*H*PO: Thrombopoietin gene
MPL: Myeloproliferative leukemia
